# A Hybrid Web-Based and In-Person Self-Management Intervention Aimed at Preventing Acute to Chronic Pain Transition After Major Lower Extremity Trauma: Feasibility and Acceptability of iPACT-E-Trauma

**DOI:** 10.2196/10323

**Published:** 2018-04-30

**Authors:** Mélanie Bérubé, Céline Gélinas, Nancy Feeley, Géraldine Martorella, José Côté, G Yves Laflamme, Dominique M Rouleau, Manon Choinière

**Affiliations:** ^1^ Centre intégré universitaire du Nord-de-l’Île-de-Montréal, Hôpital du Sacré-Coeur de Montréal Trauma Program and Department of Nursing Montreal, QC Canada; ^2^ Ingram School of Nursing McGill University Montreal, QC Canada; ^3^ Centre for Nursing Research, Jewish General Hospital Montreal, QC Canada; ^4^ College of Nursing Florida State University Tallahassee, FL United States; ^5^ Faculté des sciences infirmières Université de Montréal Montreal, QC Canada; ^6^ Centre de recherche du Centre hospitalier de l’Université de Montréal Montreal, QC Canada; ^7^ Centre intégré universitaire du Nord-de-l’Île-de-Montréal, Hôpital du Sacré-Coeur de Montréal Department of Surgery Université de Montréal Montreal, QC Canada; ^8^ Department of Anesthesiology Université de Montréal Montreal, QC Canada

**Keywords:** Acute pain, chronic pain, wound and injuries, lower extremity, self-care, health promotion, feasibility studies, patient acceptance of health care

## Abstract

**Background:**

A transition from acute to chronic pain frequently occurs after major lower extremity trauma. While the risk factors for developing chronic pain in this population have been extensively studied, research findings on interventions aiming to prevent chronic pain in the trauma context are scarce. Therefore, we developed a hybrid, Web-based and in-person, self-management intervention to prevent acute to chronic pain transition after major lower extremity trauma (iPACT-E-Trauma).

**Objective:**

This study aimed to assess the feasibility and acceptability of iPACT-E-Trauma.

**Methods:**

Using a descriptive design, the intervention was initiated at a supra-regional level-1 trauma center. Twenty-eight patients ≥18 years old with major lower extremity trauma, presenting with moderate to high pain intensity 24 hours post-injury were recruited. Feasibility assessment was two-fold: 1) whether the intervention components could be provided as planned to ≥80% of participants and 2) whether ≥80% of participants could complete the intervention. The rates for both these variables were calculated. The E-Health Acceptability Questionnaire and the Treatment Acceptability and Preference Questionnaire were used to assess acceptability. Mean scores were computed to determine the intervention’s acceptability.

**Results:**

More than 80% of participants received the session components relevant to their condition. However, the Web pages for session 2, on the analgesics prescribed, were accessed by 71% of participants. Most sessions were delivered according to the established timeline for ≥80% of participants. Session 3 and in-person coaching meetings had to be provider earlier for ≥35% of participants. Session duration was 30 minutes or less on average, as initially planned. More than 80% of participants attended sessions and <20% did not apply self-management behaviors relevant to their condition, with the exception of deep breathing relaxation exercises which was not applied by 40% of them. Web and in-person sessions were assessed as very acceptable (mean scores ≥3 on a 0 to 4 descriptive scale) across nearly all acceptability attributes.

**Conclusions:**

Findings showed that the iPACT-E-Trauma intervention is feasible and was perceived as highly acceptable by participants. Further tailoring iPACT-E-Trauma to patient needs, providing more training time for relaxation techniques, and modifying the Web platform to improve its convenience could enhance the feasibility and acceptability of the intervention.

**Trial Registration:**

International Standard Randomized Controlled Trial Number (ISRCTN): 91987302; http://www.controlled-trials.com/ISRCTN91987302 (Archived by WebCite at http://www.webcitation.org/6ynibjPHa)

## Introduction

### Background

Most trauma patients suffer from an orthopedic injury [1,2] resulting in a high prevalence of disabling chronic pain affecting up to 86% of patients from several months to years post-trauma [3-5]. Considering the negative impacts of chronic pain on the quality of life of trauma patients [3,5-8] and associated social expenditure [9-13], several studies have focused on risk factors that could trigger acute to chronic pain transition in this population [3-5]. Some risk factors have been consistently identified across studies, including moderate to high acute intensity pain, major lower extremity trauma (ET; ie, patients who usually require hospitalization for surgical and multidisciplinary team acute care management), and psychological variables (eg, anxiety, depression, pain catastrophizing, pain-related fear).

Despite a growing acknowledgment of the issues associated with chronic pain in orthopedic trauma and evidence on identified risk factors, intervention studies aiming to prevent chronic pain in this population are still scarce [14,15]. Indeed, most studies on chronic pain preventive interventions have been conducted in back pain patients [16-27] and, more recently, in the context of nontrauma related major surgery [28]. These preventive interventions were designed according to a cognitive-behavioral approach, where the objective is to promote self-management behaviors, ie, skills to control pain and its effect on physical and psychological functioning [29,30]. Preliminary findings on the efficacy of these interventions showed promising results. These included decreased pain intensity and/or disability [17,18,24-26,28], reduced opioid use [28], as well as improved psychological well-being [17,22,25] or more rapid return to work [16,19-21,27]. Hence, we developed a self-management intervention aimed at preventing acute to chronic pain transition in major lower extremity trauma (iPACT-E-Trauma) patients [31,32], a population at high-risk of developing chronic pain.

The iPACT-E-Trauma was developed according to a systematic approach, to address common factors involved in the transition from acute to chronic pain and meet the needs of patients with major lower ET [31,32]. We used empirical evidence from prior research on chronic pain preventive interventions, the biopsychosocial model of chronic pain [33], and clinical knowledge of the population to determine the main features of iPACT-E-Trauma (ie, what, who, how, where, when and how much) [34]. Then, acceptability was tested with ten clinicians (ie, nurses, orthopedic surgeons, a psychiatrist, a family physician specialized in pain management, and physiotherapists) from interdisciplinary trauma teams followed by 6 ET patients who received the intervention [32]. Both clinicians and patients found the preliminary features of iPACT-E-Trauma to be acceptable. Nonetheless, refinements were made to the intervention based on the results of an acceptability questionnaire, data gathered during a focus group with clinicians, and individual interviews with patients. Findings from the acceptability questionnaire were presented to clinicians during the focus group with them, which allowed the identification of the refinements needed. The clinicians underscored the need to improve the intervention’s suitability for ET patients. To this end, the complexity of proposed activities and session duration were reduced, making the intervention more likely to be adhered to by participants. Also, clinicians proposed to develop web sessions to facilitate the delivery of the intervention by busy health care professionals during patient’s hospitalization. The patients’ acceptability assessment highlighted the importance of tailoring the activities and timelines according to their pain intensity, pain interference with activities, implementation of self-management behaviors, and recovery pace.

The aims of this study were the following: 1) evaluate the refined version of iPACT-E-Trauma feasibility, and 2) examine its acceptability in patients with major lower ET. Feasibility and acceptability criteria as described by Sidani and Braden [35] were used in this study. Feasibility refers to the practicality of implementing the intervention, focusing on the capability to carry out components and activities as planned and identifying issues in the implementation of the intervention. Variations in implementation can occur at different levels, either with the interventionist or with the clients receiving it and who are expected to carry out recommendations in their day-to-day life [35]. Acceptability is the perceived value or attitude toward the intervention by the client. This is operationalized in different ways. First, the extent to which the intervention is effective and appropriate in addressing the presenting problem, second, whether it is convenient and poses minimal risk, and third, whether participants are willing to adhere to the intervention [35].

## Methods

### Design

A descriptive design was used. The participants were patients who received the intervention and were randomly assigned to the experimental group of a pilot randomized clinical trial (RCT) [31].

### Setting

The intervention was initiated at a 554 beds supra-regional level-1 trauma center in Montreal, Canada. This center admits, on average, 1400 trauma patients annually, 400 of who have a major ET. Patients received intervention sessions during their hospital stay, and, after hospital discharge, in a rehabilitation center, at home, or during their surgical follow-up appointment at the outpatient orthopedic clinic. Ethics approval was obtained from the Centre Intégré Universitaire de Santé et de Services Sociaux du Nord de l’île-de-Montréal, Installation Hôpital du Sacré-Coeur de Montréal (HSCM) Research Ethics Board (REB) (project identification number HSCM-2017-1333) and McGill University REB (project identification A02-M15-16B). Written consent was obtained from each participants included in the study.

### Sample Characteristics

Twenty-eight patients received the iPACT-E-Trauma intervention. The inclusion criteria were the following: a) age 18 years or older, b) able to read and speak French, (c) major lower ET, and d) at risk of developing chronic pain. Acute pain intensity has consistently been reported as a risk factor for a transition from acute to chronic pain in the ET population [3-5]. The Initiative on Methods, Measurement, and Pain Assessment in Clinical Trials has recommended the inclusion of this criterion in chronic pain prevention studies [36]. Consequently, patients were enrolled if they manifested a pain intensity of ≥4/10 upon movement 24 hours post-injury, which corresponds a moderate to severe pain intensity [37], as documented by nurses in medical charts.

The exclusion criteria were the following: a) spinal cord injury, b) amputation, c) other trauma associated with high-intensity pain (>2 fractured ribs [38] or surgical abdominal trauma) or principal site of pain not being lower ET, d) cognitive impairment and language limitation (ie, dementia, moderate-severe traumatic brain injury - Glasgow coma scale score <13/15 [39], administration of sedative agents, mechanical ventilation) affecting the capacity to participate in the intervention and to complete questionnaires, and e) needing more than 7 days of hospitalization before being eligible to participate in the study. Patients with pre-injury somatic pain were not excluded unless they were taking analgesics on a daily basis, and neither were patients with pre-injury visceral pain, considering that it is possible to differentiate this type of pain from musculoskeletal pain. Moreover, although substance abuse, including pre-injury opioid use, may influence pain outcomes, we did not exclude patients with this comorbid factor considering its high incidence in the trauma population [40-43] and the potential threat to the study’s external validity.

### Intervention

The main features of the intervention have been previously described [31,32]. The topics of the refined version of iPACT-E-Trauma were the bio-psychosocial dimensions of pain, pharmacological (including how to reduce opioids over time) and nonpharmacological strategies for acute pain management, health-promotion strategies, and return to pre-injury activities. Various strategies commonly utilized in interventions based on a cognitive-behavioral approach [44] were used, such as psychoeducation, continued monitoring, provision of feedback, problem-solving, individualized action plan for a progressive increase in activity, and matching of self-management skills with real-life situations.

**Figure 1:**
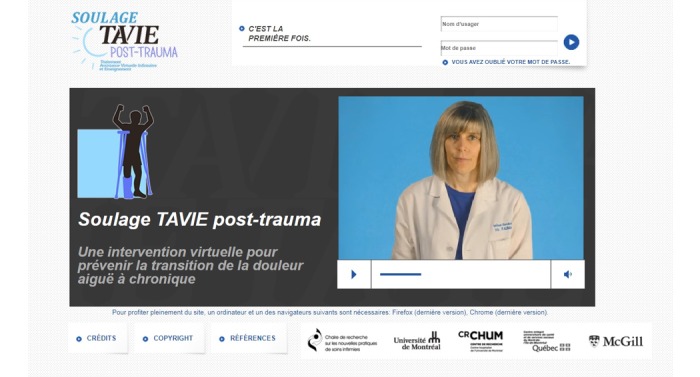
Introduction page of Soulage TAVIE Post Trauma.

**Figure 2:**
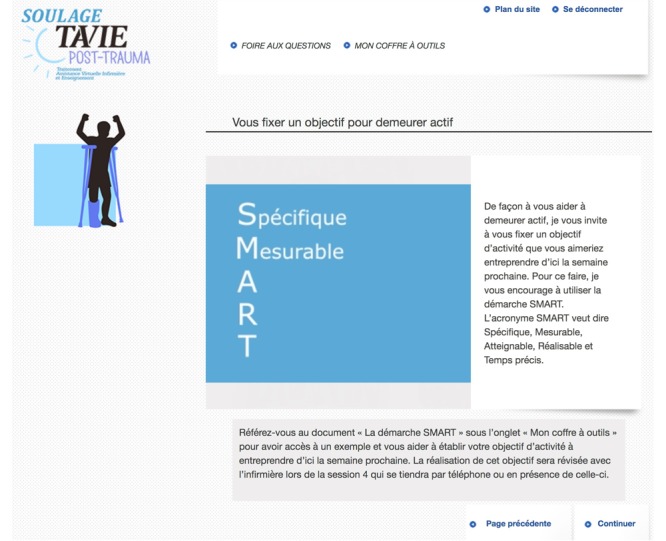
Establishing an objective for staying active after the injury.

Regarding structure, the refined iPACT-E-Trauma included seven sessions (five regular and two boosters) lasting between 15 and 30 minutes, provided by a nurse with a master’s degree [31,32]. The intervention lasted three months and was initiated within seven days post-injury to allow patients to rapidly develop self-management behaviors and optimally manage their acute pain. Sessions 1 and 2 were expected to be given in the first week post-injury, sessions 3 to 5 on a weekly basis after that, and sessions 6 and 7 at six and twelve weeks post-injury, respectively. A hybrid delivery mode was utilized combining the Web (ie, *Traitement et Assistance Virtuelle Infirmière et Enseignement* platform - Soulage TAVIE Post-Trauma) [45,46] (Figures 1 and 2; Multimedia Appendix 1) and in-person contact with a nurse, over the phone or face-to-face in the outpatient orthopedic clinic. The first three sessions were Web-based, followed by short in-person coaching meetings during hospitalization. Web sessions were delivered with a laptop and headphones in participant room. The last four sessions were designed to be one-on-one, either in a rehabilitation setting, an outpatient orthopedic clinic, a home, or a hospital in case of a lengthy hospital stay. A participant manual was used as a support tool during in-person sessions. Web sessions and the participant manual were designed according to recommended health literacy strategies (Multimedia Appendix 2) [47,48].

### Variables and Measurement Tools

Sociodemographic and clinical data were collected after participants agreed to take part in the study. A clinical profile form was used to gather data related to injuries, treatments received, and pre-injury comorbid factors. Substance abuse was determined according to the toxicology screen as well as the health questionnaire obtained soon after the arrival to the trauma center. The feasibility and acceptability of the intervention were assessed with the following tools.

#### Feasibility

Intervention feasibility was assessed according to two criteria: 1) the ability to deliver the intervention as planned (ie, provision of session components to ≥80% of participants, length of sessions corresponded to planned duration, and the challenges faced during intervention delivery could be overcome), and 2) the capability of participants to complete the intervention (ie, attendance at sessions as well as application of self-management behaviors after sessions 1 to 6 ≥80% of participants) [49]. We used an Intervention Feasibility Evaluation Logbook to document the delivery of session components and a Self-Management Behavior Assessment Checklist to describe participant’s capability to complete the intervention.

#### Acceptability

Web sessions were assessed with an E-Health Acceptability Questionnaire that includes recommended features for internet-based interventions [50], and in-person sessions were assessed with an acceptability questionnaire based on the Treatment Acceptability and Preference (TAP) Questionnaire [51]. The E-Health Acceptability Questionnaire was developed to analyze the TAVIE platform content [50], and includes 21 items rated on a 5-point descriptive scale (eg, 0 = not easy to use, 4 = very much easy to use) divided into nine subscales: ease of use, ease of understanding, credibility, tailoring, relevance, perceived applicability, visual design appreciation, dosage, motivational appeal, and overall satisfaction with the Web-based intervention. Content validation for this questionnaire was established by experts in the field of Web-based health interventions [50]. Participants completed the E-Health Acceptability Questionnaire after session 3. A high-reliability score (Cronbach alpha = 0.87) was obtained for the E-Health Acceptability Questionnaire in this study.

The TAP Questionnaire is a validated and reliable tool for persons receiving self-management interventions [51], that assesses the following intervention acceptability attributes: 1) perceived effectiveness in managing the problem, 2) appropriateness, 3) suitability to individual context, and 4) convenience or willingness to apply and adhere to the intervention. Participants were instructed to rate the intervention’s features based on these four attributes, using a 5-point descriptive scale (eg, 0 = not appropriate, 4 = very much appropriate). Open-ended questions were added at the end of each attribute section to gather input on the modifications required to improve intervention acceptability. Participants completed the TAP questionnaire after session 5 to assess the acceptability of sessions 4 and 5, and after session 7 to assess sessions 6 and 7 as well as the intervention overall. Reliability scores for acceptability questionnaires completed after sessions 5 and 7 were high when considering all four attributes (Cronbach alpha >0.9).

### Data Analysis

#### Feasibility

To determine the ability to deliver iPACT-E-Trauma, uptake of the various components (face-to-face contacts, Web pages, and on-line documents consulted) was described. Rates of sessions delivered within the established timeline were computed. Mean scores were calculated for the time spent watching Web sessions, consulting Web pages and for the delivery of in-person sessions. Descriptive data about the challenges involved in the delivery of interventions were grouped into categories. Frequencies were calculated for each category. Rates of attendance to sessions and application of self-management behaviors were calculated regarding the capability of participants to complete the intervention.

#### Acceptability

Descriptive analyses of data for the acceptability questionnaires were performed. Mean scores were calculated for each acceptability attribute. The answers to the open-ended questions on the modifications required to enhance the intervention’s acceptability were grouped into categories. However, less than five participants answered the open-ended questions, precluding meaningful data analysis.

Extracts from the data sets and/or analyzed and the material used during the current study are available from the corresponding author.

## Results

### Sociodemographics

Sociodemographic data are presented in Table 1. More than half the participants were male, and the majority were Caucasian. Mean age was 47 years, ranging from 18 to 79 years. Twenty-two out of 28 participants (78%) had a high school to college education, and 20 participants (72%) had an annual income < $ 50,000. The most common occupation was laborer followed by work as a professional. Six participants (22%) were retired.

### Clinical Data

Data on participants’ injuries and treatments are presented in Table 2. Almost half of the participants suffered an orthopedic injury secondary to a fall. The most frequent fractures were to the pelvis, the acetabulum, the femur and the tibia. Joint dislocation occurred in 13 out of 22 participants (46%) and soft tissue injury (eg, tissue swelling delaying surgery, deep laceration, crush injury) in more than half of the participants. Almost two-thirds of the participants had at least two fractures, while half had a concomitant injury. The most frequent being a fracture to the upper extremities, followed by a fracture to the spine, and mild TBI. According to the mean Injury Severity Score (ISS) and the Abbreviated Injury Scale (AIS) - Extremity score [52] most participants suffered moderate to serious injury. The dominant comorbidities were substance abuse and mental health issues (eg, history of anxiety or depression) but were present in less than a quarter of participants. Twenty-six participants (93%) had an open reduction and internal fixation surgery for their lower ET and, among these, 11 participants (39%) had a lower limb immobilized by a cast or an orthosis for several weeks after the injury. Weight-bearing limitation on the injured limb was prescribed for 3 to 6 months in almost half the participants.

**Table 1:** Sociodemographic data for total participants (n=28).

Characteristics		iPACT-E-Trauma group, n (% )
**Gender**		
	Male	15 (54)^a^
**Ethnical group**		
	Caucasian	23 (82)
	Haitian	3 (11)
	Arabic	2 (7)
**Level of education**		
	< High school diploma	2 (7)
	High school diploma	11 (39)
	Collegial diploma	11 (39)
	Undergraduate studies diploma	3 (11)
	Graduate studies diploma	1 (4)
**Occupation**		
	Laborer	6 (22)
	Clerical work	2 (7)
	Administration	4 (14)
	Professional	4 (14)
	Student	2 (7)
	None	4 (14)
	Retired	6 (22)
**Annual income**		
	< $20,000/year	6 (22)
	$20,000 to $49,000	14 (50)
	$50,000 to $69,000	2 (7)
	$70,000 to $99,000	4 (14)
	≥ $100,000	2 (7)

^a^Mean age (range)=47 (18 to 79).

### Feasibility

#### The Ability to Deliver the Intervention as Planned

Twenty to 28 out of 28 participants (71% to 100%) accessed all Web pages of sessions 1 to 3 (Table 3). During session 2, six participants (21%) did not access Web pages about the mechanisms of action of opioids and acetaminophen, and 19 participants (67%) did not access Web pages related to pregabalin. Most participants consulted self-management recommendation summaries in the participant’s manual, while a few consulted them in the Web platform throughout Web sessions.

Components of the in-person coaching meetings relevant to all participants were provided as planned to most during the first and the second meetings, and to fewer participants during the third meeting (Table 3). Those that required individualized tailoring were also less frequently delivered. Web sessions were primarily delivered according to the established timeline, except for session 3. The timeline was less frequently followed for the in-person meetings compared to Web sessions. Mean duration for Web sessions combined with in-person coaching meetings were ≤ 30 minutes. The challenges experienced during Web sessions were from various types, but they all occur in seven or less (≤ 26%) of participants: 1) environmental (ie, noise or limited space in participant’s room), 2) technical (ie, slow internet connection, difficulty creating password), 3) participant-related (ie, drowsiness, nausea, no glasses), and 4) care-related (ie, interruptions for nursing evaluation and intervention or diagnostic tests).

**Table 2:** Participants’ injuries and treatments received (n=28).

Characteristics		Results, n (%)
**Trauma mechanism**		
	Motor vehicle crash	8 (28)
	Pedestrian collision	3 (11)
	Fall	13 (46)
	Sport	3 (11)
	Work	1 (4)
**Types of orthopedic injuries^a^**		
	Pelvic fracture	12 (43)
	Acetabulum fracture	9 (34)
	Femur fracture	8 (28)
	Knee joint ligaments sprain	2 (7)
	Tibia fracture	8 (29)
	Fibula fracture	7 (25)
	Ankle fracture	5 (18)
	Foot fracture	4 (14)
	Open fracture	3 (11)
	Joint dislocation	13 (46)
	Soft tissue	16 (57)
**Number of fractures**		
	One	10 (36)
	Two	11 (39)
	≥3	7 (25)
**Other injuries**		
	Participants with at least one concomitant injury	14 (50)
	Mild traumatic brain injury	4 (14)
	Upper extremities	6 (22)
	Thorax	2 (7)
	Abdomen	3 (11)
	Spine	5 (18)
Injury Severity Score		9.4 (6)
**Abbreviated Injury Scale (AIS) – Orthopedic score**		
	AIS 1 (minor extremity injury)	—
	AIS 2 (moderate extremity injury)	15 (54)
	AIS 3 (serious extremity injury)	11 (39)
	AIS 4 (severe extremity injury, life-threatening)	2 (7)
**Comorbidities**		
	Substance abuse	5 (18)
	Somatic or visceral pain before the injury	1 (4)
	Mobility issue requiring technical aid	2 (7)
	Neurological (eg, epilepsy, previous stroke)	4 (14)
	Cardiovascular (eg, previous myocardial infarction, hypertension)	3 (11)
	Morbid obesity (Body Weight Index ≥35)	3 (11)
	Psychological (eg, anxiety, depression)	6 (22)
**Treatments^b^**		
	Open reduction and internal fixation surgery	26 (93)
	Closed reduction and external fixation surgery	8 (28)
	Conservative treatment (no surgery)	2 (7)
	Immobilization with a cast or an orthosis	11 (39)
**Weight bearing limitation postinjury**		
	No limitation	1 (4)
	6 weeks postinjury	14 (50)
	3 months postinjury	10 (36)
	6 months postinjury	3 (11)

^a^Some participants had more than one type of fractures.

^b^Some participants received more than one treatment.

The components of in-person sessions relevant to all participants were provided to a large proportion of participants during sessions 5 to 7 (Table 4), while those requiring individualized tailoring were delivered to fewer participants. In-person sessions were offered according to the established timeline to most of the participants. Their mean duration was also ≤ 30 minutes. Challenges experienced during sessions 4 to 7 were related to participants (ie, lack of motivation, emphasizing other problems than pain), to care (ie, difficulty coordinating sessions with other interventions occurring at the outpatient orthopedic clinic), and to the environment (ie, noise in participant’s room). These were present for less than three participants (< 10%).

### The Capability of Participants to Complete the Intervention

#### Attendance at the Intervention Sessions

The Web sessions and in-person coaching meetings were attended by all participants for the first two sessions and by 26 out of 28 participants (93%) for the third session (Table 5). The in-person sessions were attended by all participants for session 4, by 26 (93%) for sessions 5 and 6, and by 25 (89%) for session 7 (Table 5).

#### Application of Self-Management Behaviors

Overall, less than six over 28 participants (< 20%) did not apply self-management behaviors relevant to their condition (Table 5). Cryotherapy was applied by two-thirds of participants after session 1 and by more than half after session 3 and diminished as the intervention progressed. Cryotherapy was not indicated in several participants after sessions 1 to 4 given they had limb immobilization with a thick elastic bandage, splint cast with an elastic bandage or skin vascularization issues. Following sessions 5 and 6, cryotherapy was not indicated in 18 participants (70%) because pain intensity did not interfere with activities, there was no significant limb swelling, or a splint covered by a thick elastic bandage immobilized the limb. Leg elevation, a self-management behavior suggested in the first session, was strongly followed from sessions 1 to 3, and as was the case with cryotherapy, its use gradually declined afterward. Leg elevation was not needed in some participants after sessions 1 and 2, considering localized pelvic fractures without associated swelling, and even more after sessions 3 to 7 (reaching up to 20 participants or more than 75% of them) as the gradual decrease in swelling and pain intensity helped participants resume activities.

Appropriate use of co-analgesia was implemented by all but one participant after session 1 and in about two thirds after session 4, after which co-analgesia was not needed in up to 44% of participants, as they were either only taking acetaminophen or no analgesic. Almost half the participants did not use the deep breathing relaxation exercises they were taught in session 2. Relaxation exercises were not indicated anymore for many participants after sessions 4 to 7 because there was no marked pain interference with activities (score<4/10) [37]. Problem-solving, when facing a difficult pain experience, was used by 10 participants (36%) after session 3 and three participants (10%) after session 6. However, this self-management behavior did not apply to many participants and was found irrelevant. Moreover, few participants needed to establish sleep hygiene objectives and apply strategies to facilitate sleep over the course of the intervention sessions.

The objective of remaining active without increasing pain intensity and the individualized plans for returning to previous activities were highly achieved by participants. Mobility restrictions prevented some participants from reaching their objectives for staying active. Regarding strategies, several participants used the gradual return to activities, while fewer participants used activity pacing or changing the activity schedule in light of pain intensity variations throughout the day. These two latter strategies were not indicated for several participants since they were not yet active enough.

**Table 3:** Delivery of web sessions (1 to 3) and related in-person coaching meetings.

Variables						Results
**Session 1**						
	**Web (n=28), n (%)**					
		Participants who accessed all web pages				28 (100)
		Summaries accessed in the web platform				4 (14)
		Summaries consulted in the participant manual				24 (85)
		Session delivered according to the established timeline				28 (100)
		Session duration, mean (SD; range)				18 min (6; 13-34)
	**In-person coaching (n=28)**					
		**Components provided to participants, n (%)**				
				Answer questions related to the on-line content		27 (96)
				Ask participants to report their pain intensity		28 (100)
				Ask participants to report their ice and legs elevation utilization		28 (100)
				Review how to use ice and legs elevation if needed		12 (46)
				Tailor the recommendations on cryotherapy and legs elevation if needed		17 (61)
		Meeting delivered according to the established timeline, n (%)				12 (43)
		Meeting duration, mean (SD; range)				4 min (2; 2-10)
**Session 2**						
	**Web (n=28), n (%)**					
		Participants who accessed all web pages				20 (71)
		Summaries accessed in the web platform				4 (14)
		Summaries consulted in the participant manual				24 (86)
		Session delivered according to the established timeline				27 (96)
		Session duration, mean (SD; range)				20 min (7.4; 11-47)
	**In-person coaching (n=28)**					
		**Components provided to participants, n (%)**				
				Answer questions related to the on-line content		27 (96)
				Ask participants to report their pain intensity		27 (96)
				Ask participant to report their co-analgesia, ice and legs elevation utilization		28 (100)
				Review how to use co-analgesia, relaxation exercises ice and legs elevation		16 (57)
				Tailor the recommendations on co-analgesia if needed		4 (14)
				Tailor the recommendations on cryotherapy and legs elevation if needed		8 (29)
		Meeting delivered according to the established timeline, n (%)				16 (57)
		Meeting duration, mean (SD; range)				6 min (3; 2-15)
**Session 3**						
	**Web (n=26), n (%)**					
		Participants who accessed all web pages				25 (96)
		Summaries accessed in the web platform				2 (8)
		Summaries consulted in the participant manual				24 (92)
		Session delivered according to the established timeline				17 (65)
		Session duration, mean (SD; range)				16 min (4; 8-33)
	**In-person coaching (n=26)**					
		**Components provided to participants, n (%)**				
				Answer questions related to the on-line content		16 (62)
				Ask participants to report their pain intensity		13 (50)
				Ask participants to report their co-analgesia and relaxation exercises utilization		14 (54)
				Review how to use co-analgesia and relaxation exercises if needed		10 (39)
				Tailor the recommendations on co-analgesia if needed		5 (19)
				Invite participants to discuss the use of problem solving if indicated		9 (35)
				Assist participants in the establishment of an activity objective		26 (100)
		Meeting delivered according to the established timeline, n (%)				4 (15)
		Meeting duration, mean (SD; range)				8 min (6; 2-23)

#### Acceptability

Web sessions were assessed as very acceptable nearly across all acceptability attributes (Table 6). Visual appeal (ie, colors, pictures, and pages outlook) and applicability (ie, perceived capacity to apply strategies recommended in Web sessions) were rated as acceptable, on average. In-person weekly sessions 4 and 5 were also assessed by participants as very acceptable for almost every attribute (Table 7). Items that were evaluated as acceptable included the perceived effectiveness of establishing an individualized action plan for returning to pre-injury activities, defining objectives to achieve adequate sleep hygiene, and reviewing previously learned self-management strategies at the beginning of each session. In-person sessions 6 and 7 (ie, booster sessions) were assessed as acceptable to very acceptable (Table 7). Items with the lowest mean scores across acceptability attributes were the following: reviewing the individualized action plan to return to pre-injury activity and establishing a new action plan, as well as the convenience of phone sessions.

**Table 4:** Delivery of in-person sessions (4 to 7).

Variables			Results
**Session 4: in-person (n=28)**			
	**Components provided to participants, n (%)**		
		Ask participants to report their pain intensity	27 (96)
		Ask participants to report their analgesics utilization	28 (100)
		Encourage the application of learned self-management behaviors if needed	20 (71)
		Provide information on gradual reduction of analgesics if needed	10 (36)
		Discuss the use of problem solving if indicated	11 (39)
		Provide feedback on the achievement of activity objective	26 (93)
		Offer assistance in the establishment of another activity objective	24 (86)
		Provide information on sleep hygiene	28 (100)
		Provide assistance in the establishment of a sleep hygiene objective if needed	9 (32)
		Encourage the use of strategies to optimize sleep if needed	14 (50)
	Session delivered according to the established timeline, n (%)		26 (93)
	Session duration, mean (SD; range)		19 min (7; 8-38)
**Session 5: in-person (n=26)**			
	**Components provided to participants, n (%)**		
		Ask participants to report their pain intensity	26 (100)
		Ask participants to report their analgesics utilization	26 (100)
		Encourage the application of learned self-management behaviors if needed	18 (69)
		Provide information on gradual reduction of analgesics utilization if needed	8 (31)
		Provide feedback on the achievement of sleep hygiene objective	8 (31)
		Encourage the continuous use of strategies to optimize sleep if needed	13 (50)
		Providing feedback on the achievement of activity objective	23 (89)
		Give information on how to return to pre-injury activities if needed	16 (64)
		Provide assistance for establishing a plan for returning to pre-injury activities	25 (93)
	Session delivered according to the established timeline, n (%)		24 (92)
	Session duration, mean (SD; range)		20 min (6; 12-31)
**Session 6 (Booster 1): in-person (n=26)**			
	**Components provided to participants, n (%)**		
		Answer questions related to pain management strategies	13 (50)
		Ask participants to report their analgesics utilization	26 (100)
		Give information on gradual reduction of analgesics if needed	8 (31)
		Provide feedback on action plan achievement	26 (100)
		Provide assistance for reviewing the plan for returning to pre-injury activities	26 (100)
		Reinforce the importance of using learned self-management behaviors to facilitate the return to pre-injury activities if needed	10 (77)
	Session delivered according to the established timeline, n (%)		22 (85)
	Session duration, mean (SD; range)		18 min (8; 7-50)
**Session 7 (Booster #2): in-person (n=25)**			
	**Components provided to participants, n (%)**		
		Answer questions related to pain management strategies	14 (56)
		Ask participants to report their analgesics utilization	22 (88)
		Give information on gradual reduction of analgesics utilization if indicated	6 (24)
		Providing feedback on action plan achievement	25 (100)
		Provide assistance for reviewing the plan for returning to activities	24 (96)
		Reinforce the importance of using learned self-management behaviors to facilitate the return to pre-injury activities if required	19 (76)
	Session delivered according to the established timeline, n (%)		24 (96)
	Session duration, mean (SD; range)		15 min (5; 10-30)

**Table 5:** Intervention completion by participants (N=28).

Variables					Applied, n (%)		Not applied as recommended or not applied, n (%)		Not indicated, n (%)	
**Session 1 (n=28)**										
	**Behaviors applied between session 1 and 2**									
		Cryotherapy (every 2h for 20 min)				17 (61)		4 (14)		7 (25)
		Legs elevation in straight position while in bed				24 (86)		1 (3)		3 (11)
**Session 2 (n=28)**										
	**Behaviors applied between session 2 and 3**									
		Cryotherapy				18 (64)		3 (11)		7 (25)
		Legs elevation in straight position				24 (86)		2 (7)		2 (7)
		Co-analgesia				27 (96)		—^a^		1 (4)
		Breathing relaxation exercises when experiencing pain interference with activities				11 (39)		11 (39)		6 (21)
**Session 3 (n=26)**										
	**Behavior applied between session 3 and 4^b^**									
		Cryotherapy				16 (57)		5 (18)		7 (25)
		Legs elevation in straight position				18 (64)		3 (11)		7 (25)
		Co-analgesia				22 (79)		2 (7)		4 (14)
		Breathing relaxation exercises				8 (29)		12 (43)		8 (29)
		Problem solving				10 (36)		5 (18)		13 (46)
		Implementation of the activity objective				24 (86)		1 (4)		3 (11)
		Gradual return to activities				25 (89)		—		3 (11)
		Changing schedule of activities in light of pain				5 (18)		4 (14)		19 (68)
		Activity pacing				15 (57)		1 (3)		11 (39)
**Session 4 (n=28)**										
	**Behavior applied between session 4 and 5^c^**									
		Co-analgesia (with reduction of opioids)				17 (65)		1 (4)		8 (31)
		Problem solving				10 (39)		1 (4)		15 (58)
		Implementation of the activity objective				20 (77)		5 (19)		1 (4)
		Gradual return to activities				21 (81)		1 (4)		4 (15)
		Changing schedule of activities in light of pain				3 (12)		1 (4)		22 (85)
		Activity pacing				18 (69)		—		8 (31)
		Implementation of sleep hygiene objective				8 (31)		2 (8)		16 (62)
		Strategies to facilitate sleep				10 (39)		—		16 (62)
		**Other pain management strategies**								
				Breathing relaxation exercises		4 (15)		6 (23)		16 (62)
				Cryotherapy		12 (46)		1 (4)		13 (50)
				Legs elevation		13 (50)		—		13 (50)
**Session 5 (n=26)**										
	**Behaviors applied between session 5 and 6**									
		Co-analgesia (with reduction of opioids)				17 (65)		1 (4)		8 (31)
		Implementation of the action plan				24 (92)		2 (8)		—
		Gradual return to activities				18 (69)		3 (12)		5 (19)
		Changing schedule of activities in light of pain				3 (12)		21 (81)		2 (8)
		Activity pacing				17 (65)		1 (4)		8 (31)
		**Other pain management strategies**								
				Breathing exercises		1 (4)		5 (19)		20 (77)
				Cryotherapy		8 (31)		—		18 (69)
				Legs elevation		5 (19)		1 (4)		20 (77)
				Strategies to facilitate sleep		3 (12)		—		23 (89)
				Problem solving		7 (27)		2 (8)		17 (65)
**Session 6 (n=26)**										
	**Behavior applied between session 6 and 7^d^**									
		Adequate use of analgesics (with no or minimal use of opioids)				14 (56)		—		11 (44)
		Implementation of the action plan				25 (96)		1 (4)		—
		Gradual return to activities				21 (84)		1 (4)		3 (12)
		Changing schedule of activity in light of pain				3 (12)		2 (8)		20 (80)
		Activity pacing				18 (72)		6 (24)		1 (4)
		**Other pain management strategies**								
				Breathing relaxation exercises		1 (4)		4 (17)		19 (79)
				Cryotherapy		4 (16)		3 (12)		18 (72)
				Legs elevation		5 (20)		3 (12)		17 (68)
				Strategies to facilitate sleep		3 (12)		—		22 (88)
				Problem solving		3 (12)		1 (4)		21 (84)

^a^The category does not apply to any participant.

^b^Percentage was calculated from 28 participants since the application of self-management behaviors was verified at the beginning of session 4.

^c^Percentage was calculated from 26 participants since the application of self-management behaviors was verified at the beginning of session 5.

^d^Percentage was calculated from 25 participants since the application of self-management behaviors was verified at the beginning of session 7.

**Table 6:** Web-based sessions (1 to 3) acceptability.

Web session components			Results, mean^a^ (SD), (n=28)
**Navigation**			
	Directives and instructions	3.4 (1.0)	
	Web pages navigation	3.5 (1.0)	
**Understanding**			
	Language and vocabulary used by the nurse	3.8 (0.5)	
	Content	3.7 (0.7)	
**Credibility**			
	Content and documents	3.4 (0.8)	
**Virtual nurse and information tailoring**			
	Appreciation of nurses’ videos	3.8 (0.5)	
	Interactions with the virtual nurse	3.8 (0.4)	
	Perception to have received a tailored consultation	3.4 (1.0)	
	Personalization of messages	3.1 (1.1)	
**Individual relevance**			
	Content and documents	3.4 (0.6)	
	Appropriateness for the management of pain and for returning to activities	3.4 (0.7)	
	Recommendations corresponding to participant’s needs	3.6 (0.6)	
	Usefulness	3.3 (0.6)	
**Applicability**			
	Capacity to implement strategies recommended in web sessions	2.9 (1.1)	
**Visual appealing**			
	Videos	3.3 (0.7)	
	Colors, pictures and pages outlook	2.8 (1.0)	
**Dosage**			
	Sessions duration	3.1 (1.1)	
	Interval of time between each session	3.1 (1.1)	
	Number of sessions	3.3 (0.8)	
**Motivational appealing**			
	The participant would recommend web sessions to patients with ET	3.7 (0.6)	
**In-person coaching session**			
	Relevance of follow-up made by the nurse between sessions	3.6 (0.6)	
	Usefulness of follow-up made by the nurse between sessions	3.5 (0.7)	
**General**			
	Global satisfaction	3.4 (0.9)	

^a^Range (0-4)

**Table 7:** In-person sessions (4 to 7) acceptability.

Intervention Components and Features		Effectiveness, mean^a^ (SD)	Appropriateness, mean (SD)	Suitability, mean (SD)	Convenience, mean (SD)
**Sessions 4 and 5 (n=25)^b^**					
	Feedback and encouragements on the utilization of recommended pain management strategies at the beginning of each session	3.1 (0.8)	3.2 (0.8)	3.2 (0.7)	3.2 (0.8)
	Review of previously learned self-management strategies at the beginning of each session according to participant’s needs^c^	2.9 (0.8)	3.0 (0.8)	3.2 (0.8)	—
	Education on sleep hygiene strategies	3.0 (1.0)	3.2 (0.9)	3.1 (1.0)	3.5 (0.8)
	Establishment of an objective to attain adequate sleep hygiene	2.7 (0.9)	3.0 (0.9)	3.0 (0.9)	3.2 (0.7)
	Guidance on the gradual reduction of analgesics utilization	3.1 (0.9)	3.1 (0.9)	3.2 (1.0)	3.2 (1.2)
	Establishment of objectives to stay active	3.0 (0.8)	3.2 (0.8)	3.1 (0.8)	3.0 (1.0)
	Discussion on problem-solving utilization	3.2 (0.9)	3.2 (0.8)	3.2 (0.8)	3.0 (0.8)
	Establishment of an action plan for returning to pre-injury activities	2.5 (1.2)	3.1 (1.0)	3.0 (1.0)	3.4 (0.7)
	The number of weeks between each session (one week)^c^	—	—	3.2 (0.8)	—
	Sessions duration^c^	—	—	3.4 (0.7)	—
**Sessions 6 and 7 (boosters; n=23)^b^**					
	Review of previously learned self-management strategies at the beginning of each session according to participant’s needs^c^	3.0 (0.8)	3.1 (0.8)	2.9 (0.9)	—
	Guidance on gradual reduction of analgesics utilization	3.0 (0.8)	3.5 (0.6)	3.2 (0.7)	3.1 (0.8)
	Review of the action plan for returning to pre-injury activities	2.7 (0.9)	2.9 (0.9)	2.9 (1.0)	3.0 (1.0)
	Establishment of a new action plan for returning to pre-injury activities	2.7 (1.0)	2.9 (0.9)	3.0 (1.0)	3.0 (0.9)
	Having received sessions over the phone^c^	—	—	3.0 (0.9)	2.8 (1.1)
	Having received sessions in-person^c^	—	—	3.5 (0.6)	3.1 (0.9)
	The number of week between each session^c^	—	—	3.1 (0.9)	—
	Sessions duration^c^	—	—	3.1 (0.8)	—
	The sequence of the topics covered during the intervention	—	—	3.3 (0.6)	—
**Intervention duration (3 months)^c^**		—	—	3.1 (0.8)	3.1 (1.0)
	The total number of sessions included in the intervention (7 sessions)	—	—	3.0 (0.7)	3.0 (1.0)

^a^Range (0-4).

^b^A total of 25 participants completed the acceptability questionnaire related to sessions 4 and 5. A total of 23 participants completed the acceptability questionnaire related to sessions 6 and 7

^c^Only relevant acceptability items were assessed

## Discussion

### Principal Findings

This study aimed to determine the feasibility and acceptability of iPACT-E-Trauma. Findings were positive for feasibility criteria, with components for Web sessions and in-person sessions provided to ≥80% of participants, except components covered in Web session 2, in-person coaching meeting 3, and those that required individualized tailoring. Sessions were delivered according to the established timeline for ≥80% of participants, excluding session 3 and in-person coaching meetings for sessions 1 to 3. Average session duration was ≤30 minutes, as expected. Moreover, except for one participant, all the challenges faced during intervention delivery were overcome, either by assisting participants with internet use or rescheduling sessions. Regarding participants’ adherence to the intervention, ≥80% were able to attend planned sessions. Likewise, most participants applied self-management behaviors relevant to their condition, except deep breathing relaxation exercises. Overall, session features were evaluated as very acceptable and no feature was considered as not acceptable.

Findings from this study highlighted ways to improve the feasibility and acceptability of iPACT-E-Trauma in preparation for a larger scale study. Additional tailoring of iPACT-E-Trauma by adjusting its content, dosage, and timing of session delivery is required to improve the ability to deliver the intervention and the capability of patients to apply self-management behaviors (ie, feasibility). Another change would be to enhance the perceived applicability of some recommended pain management strategies (ie, acceptability). Tailored interventions are based on characteristics that are unique to the person receiving it, using a combination of information or changing strategies to achieve the outcomes of interest [53,54]. The procedures to tailor self-management interventions involve increasing relevance or meaning of the content by including personally identifiable information and explaining how information is relevant to a person’s condition (ie, personalization). This also includes making recommendations related to the targeted behaviors (ie, feedback), and adapting the intervention (ie, content, dose, delivery timing) according to individual data such as determinants of the targeted behaviors [53-55]. In this study, iPACT-E-Trauma was personalized by suggesting pain management strategies relevant for patients with lower ET and by specifying in which context such strategies were applicable. Questioning patients on pain intensity, pain interference with activities, and application of self-management behaviors at each intervention session also promoted individualized feedback and content matching, according to participants’ needs.

Recent research showed that tailored Web-based and non Web-based health interventions are slightly more effective than nontailored interventions [56-59]. One of the main causes of this result is that features of tested interventions were not enough matched to the participants’ profile [55-59]. Thus, in iPACT-E-Trauma, self-management recommendations to participants should be based on behaviors they can implement considering their condition, personal attributes, and recovery pace. For example, information on how to take pregabalin should only be provided to those that use this analgesic. Problem-solving in the presence of a difficult pain experience should be exclusively reinforced in participants who experience problems regulating their negative thoughts and emotions in the presence of pain. Moreover, promoting strategies for staying active and returning to previous activities should consider the participant’s capacity to ambulate.

Concerning the dosage of iPACT-E-Trauma, the number of sessions (ie, less or more than 7 sessions) offered to participants should be tailored according to pain intensity, pain interference with activities, and abilities in pain self-management. For example, a greater number of sessions should be provided to participants who still experience significant pain interference with activities (ie, score ≥4/10) 3 months after their injury and who still need support from a health care professional for the implementation of self-management behaviors. Fewer than 7 sessions could also be offered to participants with pain intensity < 4/10 and who have restarted to ambulate on their injured limb(s).

Furthermore, the timing of in-person coaching meetings, Web session 3 and booster sessions should be revised. In-person coaching meetings were integrated between each Web-based session, since clinicians and patients emphasized the importance of keeping in direct contact with health care professionals providing the intervention during the development phase of iPACT-E-Trauma. More frequent interactions with health care professionals have also been identified as an important strategy to increase adherence to Web-based health interventions [60,61]. In-person coaching was planned 24 hours after each Web session, to give participants enough time to implement self-management behaviors. However, this study found that in-person coaching should be offered right after Web sessions to answer questions on the content covered and tailor self-management recommendations when required.

Web session 3 had to be delivered earlier than planned or was not delivered to some participants because of early hospital discharge. Also, components of the third in-person coaching meeting were not provided to each participant due to the time constraints associated with their hospital discharge. Hence, the timing of session delivery should be more flexible, to adjust to participant’s hospital length of stay. Another option would be to deliver session 3 in-person for those who do not have internet access after hospital discharge. Moreover, patients may experience less pain to their injured extremity when no weight is put on it. Hence, booster sessions, which focus on reviewing learned self-management behaviors and establishing an individualized plan for returning to previous activities, should be scheduled after participants are allowed to fully weight bear on their injured extremity. Doing so will allow participants to re-engage in self-management behaviors required to prevent pain relapse while returning to their normal activities of daily living [62]. Likewise, considering that participants preferred to receive sessions face-to-face, the timing of session delivery should be coordinated, as much as possible, with the orthopedic surgeon appointment at the outpatient clinic.

The steps necessary to further tailor iPACT-E-Trauma could be achieved through a Sequential Multiple Assignment Randomized Trial (SMART). This type of design allows the development of adaptive interventions in which the components and the dosage of the intervention are personalized, on the basis of patient characteristics or clinical presentation. They are then repeatedly adjusted over time to individual progress [63]. Adaptive interventions include a multistage process, operationalized via a sequence of decision rules that recommend when and how the intervention should be modified, in order to maximize the effects on outcomes [63]. In a SMART, participants move through multiple stages and are randomly assigned to one of several intervention options at each stage, allowing for a comparison of their efficacy [64].

Findings related to the application of self-management behaviors also indicated that the integration of relaxation therapies to iPACT-E-Trauma must be reexamined. Relaxation therapies include a number of techniques, such as progressive muscle relaxation, guided imagery, hypnosis and deep breathing exercises [65]. In this study, only deep breathing exercises were taught. Ease of implementation in the acute care context, while also providing participants with a strategy to decrease their anxiety and its effect on pain intensity, at rest and during mobilization, made this technique relevant [66]. Nevertheless, a large proportion of participants did not practice deep breathing exercises, which could be explained by the fact that relaxation techniques require training [46,67,68]. Indeed, in a recent study conducted in patients with acute orthopedic trauma, with positive disability and pain outcomes, relaxation techniques (ie, deep breathing and progressive muscle relaxation) were taught during a 60-minute session, and patients were instructed to practice daily, guided by videos [14]. Therefore, more training time should be scheduled for participants in future applications of iPACT-E-Trauma, to optimize their use of relaxation therapies. Other techniques, such as progressive muscle relaxation, could also be offered to participants, particularly for those experiencing considerable pain inference with activities.

Another improvement to iPACT-E-Trauma relates to the feasibility of using the Web platform. Some participants needed assistance to create and enter a password at the beginning of Web sessions or did not consult actionable content (eg, Web pages on the analgesics prescribed) requiring interactions from participants with the platform to access programmed information, while most participants did not consult self-management recommendation summaries integrated in a toolbox. As many as 50% of adults have limited literacy skills [69], which may affect how they find, understand, and use information on the Web. Moreover, even users with high literacy skills may find reading and using the Web more difficult when they are sick and stressed [70]. To help developers designing digital health information tools for users with limited literacy, the Office of Disease Prevention and Health Promotion of the US Department of Health and Human Services [69] has recently developed an evidence-based guide on health literacy online. Several strategies presented in this guide could be used to overcome issues faced during Web session delivery. One of these is avoiding asking users to enter too much information. Therefore, only the participant’s name could be used to access the Web sessions in iPACT-E-Trauma, since no confidential information is shared on the platform. Also, clickable elements to consult actionable content should be made more recognizable. For example, large and bright clickable buttons in a contrasting color from the surrounding text and background, and obviously clickable (eg, rectangular shape and rounded corners) could be created. Such strategies could also improve the visual appeal of the Web application, and therefore its acceptability. The summaries on self-management recommendations presented throughout Web sessions could be removed to avoid links to pages with redundant content and provided in a paper format to participants as needed.

### Study Strengths and Limitations

This study is the first to assess the feasibility and acceptability of a hybrid, Web-based and in-person, intervention for the prevention of chronic pain, to be initiated in acute care settings. Nonetheless, there are some limitations that must be addressed. First, the implementation of self-management behaviors was self-reported by participants, which could have introduced a social desirability bias in the study. To avoid this, participants were invited to discuss how they applied self-management behaviors with the interventionist at each session, instead of using a formal questionnaire, which also provided the opportunity for feedback and to determine the content that needed to be reviewed. Second, it is not possible with this study to draw any conclusions on the effect of iPACT-E-Trauma. Findings from both this study and a pilot RCT [31] in which the feasibility of the research methods will also be assessed will serve for the development of a full-scale RCT. This type of study will make it possible to determine if iPACT-E-Trauma can prevent chronic pain after a major lower ET.

### Conclusions

This study showed that iPACT-E-Trauma is feasible and perceived as highly acceptable by patients. Further tailoring the intervention, better support when learning deep breathing relaxation exercises, and modifying the Web platform to increase its convenience could improve both the delivery of iPACT-E-Trauma and patient satisfaction. Several studies have focused on the evaluation of self-management interventions when the pain has already become chronic. However, there is a pressing need for an intervention that can prevent disabling and costly chronic pain problems that often ensue after a major injury. The development of iPACT-E-Trauma is a milestone in the research efforts aimed at developing a relevant chronic pain preventive intervention that could be easily applied in the acute and rehabilitation continuums of care.

## Appendix

Multimedia Appendix 1 Screenshots of Soulage TAVIE Post Trauma.

Multimedia Appendix 2 Design of the intervention material (web sessions and participant manual) according to health literacy strategies.

## Abbreviations

AIS: Abbreviated Injury Scale
iPACT-E-Trauma: Intervention to prevent acute to chronic pain after major lower extremity trauma
ET: extremity trauma
ISS: Injury Severity Score
TAP: Treatment and acceptability preference
TAVIE: Traitement et Assistance Virtuelle Infirmière et Enseignement
