# Supplementary material for: A Hybrid Web-Based and In-Person Self-Management Intervention Aimed at Preventing Acute to Chronic Pain Transition After Major Lower Extremity Trauma: Feasibility and Acceptability of iPACT-E-Trauma
Source: JMIR Form Res. 2018 Apr 30;2(1):e10323. doi: 10.2196/10323 (PMC6334695; doi:10.2196/10323)
Supplement: Multimedia Appendix 1 [file formative_v2i1e10323_app1.pdf]

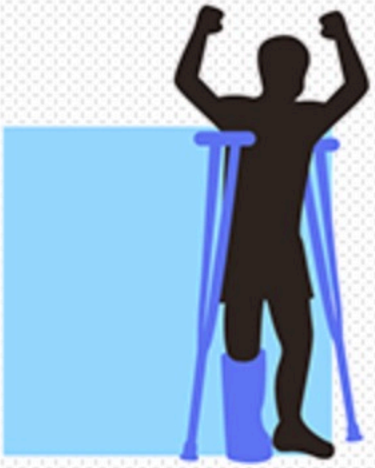

Douleur moyenne entre 4 à 10/10 ou d'intensité modérée à sévère lors de mouvements

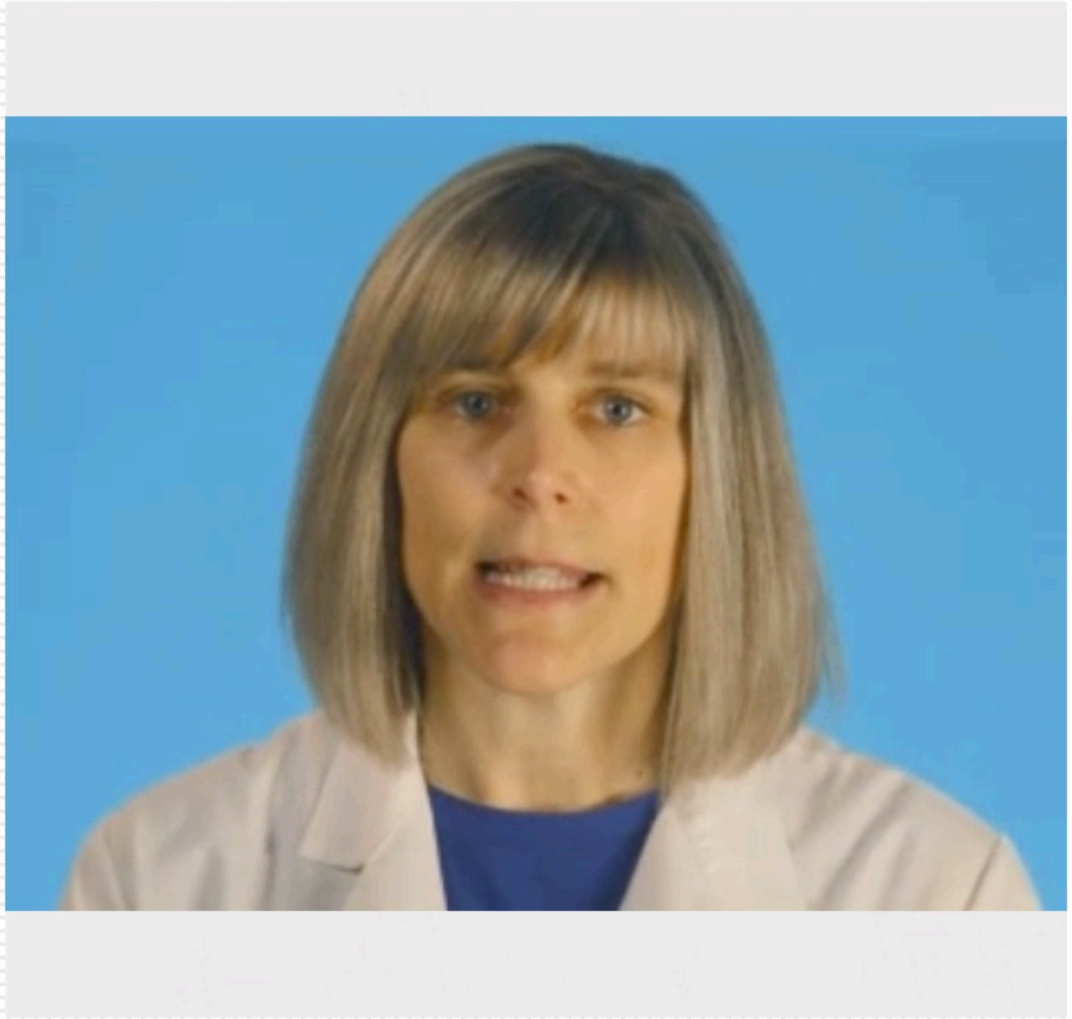

Votre réponse indique que vous avez beaucoup de douleur lors de mouvements. Il ne faut pas se décourager! Votre traumatisme est arrivé il y a peu de temps et il est normal que le fait de bouger cause à certains moments une intensité élevée de douleur. Heureusement, il existe plusieurs trucs pour soulager la douleur qui vous

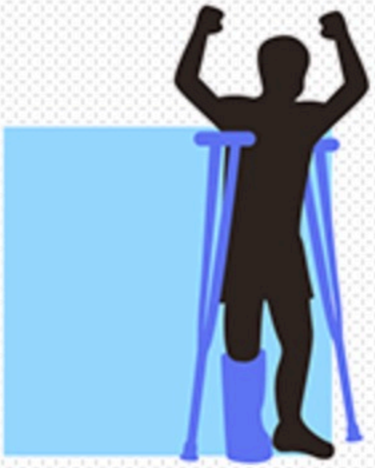

Mieux comprendre les composantes impliquées dans la douleur

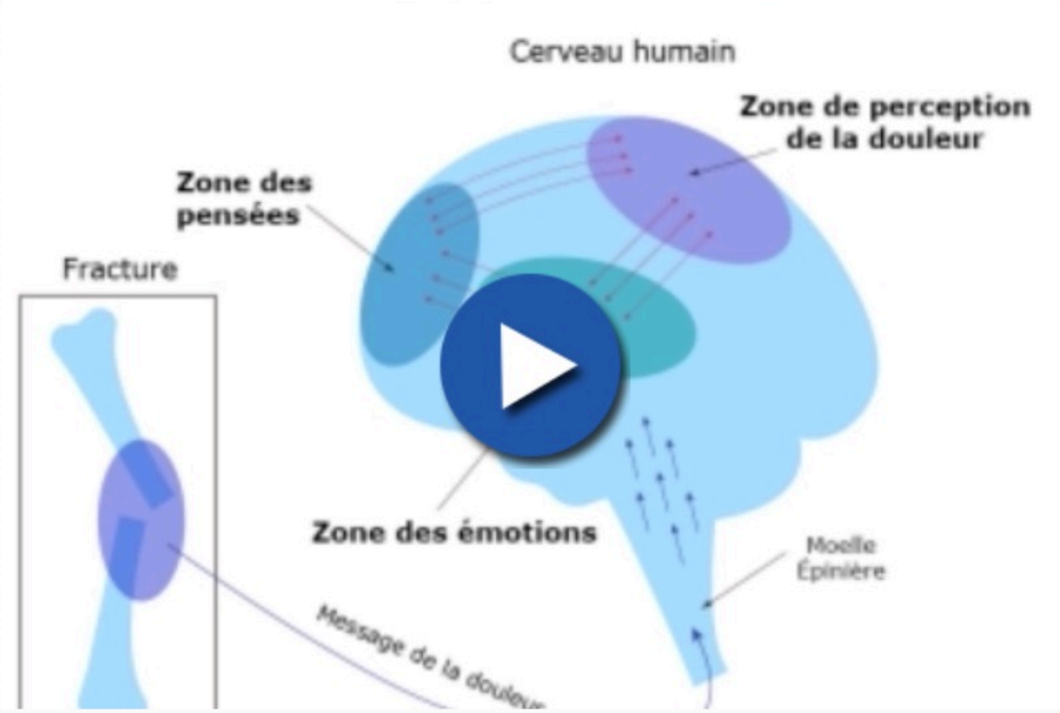

0:18 / 1:00

Il est maintenant reconnu que la douleur n'est pas seulement causée par un os cassé ou une coupure à la peau, soit des composantes physiques. L'étude du cerveau a permis de démontrer des connections entre les zones de la perception de la douleur, soit la composante physique de la douleur, et les zones des

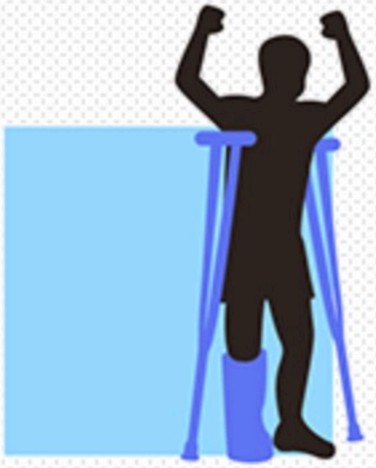

Histoire de Mathieu et l’utilisation de la glace pour soulager la douleur

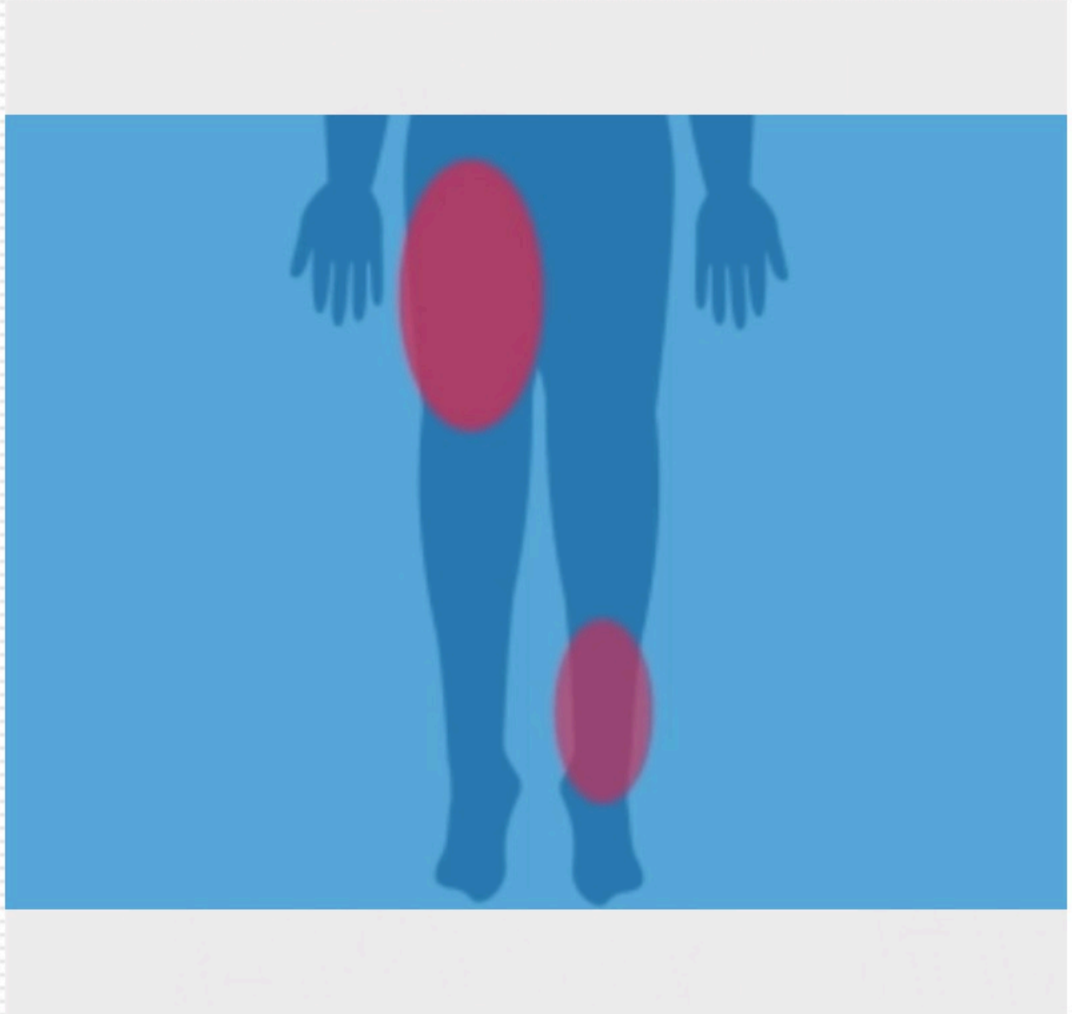

Moi c’est Mathieu. Je suis tombé à moto il y a deux jours et je me suis fracturé 2 os dans la jambe gauche et un os dans la cuisse droite. J’ai été opéré et il y un fixateur externe qui maintient mes os en place dans ma jambe gauche. Ma cuisse droite et ma cheville gauche sont très enflées. J’ai l’impression que ma peau est

Je vous invite à consulter la grille horaire de l’application de glace par Mathieu sous l’onglet « Mon coffre à outils ». Cet exemple pourra vous aider à mieux comprendre à quelle fréquence et quand appliquer la glace peu après votre traumatisme.
