# Supplementary material for: A Hybrid Web-Based and In-Person Self-Management Intervention Aimed at Preventing Acute to Chronic Pain Transition After Major Lower Extremity Trauma: Feasibility and Acceptability of iPACT-E-Trauma
Source: JMIR Form Res. 2018 Apr 30;2(1):e10323. doi: 10.2196/10323 (PMC6334695; doi:10.2196/10323)
Supplement: Multimedia Appendix 2 [file formative_v2i1e10323_app2.docx]

Design of the intervention material (web sessions and participant manual) according to

health literacy strategies

| **Health literacy strategies for web-based intervention** | **Design elements of Soulage TAVIE Post-Trauma** |
| --- | --- |
| - Determine content according to users and their goals | - The content of the web sessions (i.e., how to assess pain intensity, cryotherapy, leg elevation, analgesics, relaxation skills, emotion-thought regulation, objectives for staying active) was determined according to the pain management strategies that patients need to learn and apply promptly after a major lower ET. |
| - Write actionable content | - Web sessions are focused on self-management behaviors. A virtual nurse describes the recommended behaviors and the benefits associated to their implementation. Required behaviors are broken down into smaller steps. Checklists on these steps are available in a Toolbox of questions and in the participant manual. - A positive tone is used to keep the participant motivated. - Recommendations on self-management behaviors are listed in an active voice and the second-person pronoun is used to speak directly to the participant. |
| - Display content clearly on the page | - Each web page heading highlights the self-management behaviors that need to be learned and the steps required to apply them. Headings are formulated as questions when the participant should act. - Only one theme is covered per web page. A total of 41 web pages are included. - Sans serif fonts (i.e., Helvetica Neue and Arial) and a 14-point size are used. - Line height is 1.5 times the font size. - Sentences are kept to 20 words or less, and paragraphs to a maximum of 3 lines. Sentences are presented in list form whenever possible. - Blank spaces are used between sections and around pictures, and the content is displayed on the center of the screen. - Links are available to direct the participant towards tools and resources that supplement and support the videos and text. - Videos of real patients and illustrations are used to explain the content. Descriptive captions, explaining pictures with simple line drawings, are used. Alternative text to describe videos and illustrations are available. - Dark text on a white or very light background is used. - Web sessions are accessible using a computer screen only considering the size of videos and illustrations used on each page. |
| - Organize content and simplify navigation | - The information is conveyed using the same format in each session: 1) Session objectives, 2) Review previous teachings, 2) Assessment of pain intensity and interference with activities, 3) Presentation of the self-management behavior that needs to be mastered 4) How to apply the self-management behavior, 5) Patient storytelling, 6) Conclusions with reminders on how to apply self-management behaviors. - Simple and consistent navigation is used with large “back” and “next” buttons always positioned identically on each web page. Toolboxes and a web session glossary are available at the top of the screen on each web page. Questions that need to be answered by participants are placed at the bottom of the screen under the central portion. |
| - Engage users with interactive content | - Short videos (30 to 60 seconds) of a nurse providing educational content and feedback to patients are shown. The nurse was filmed from further away for the introduction of general information and close-up for the provision of feedback, to help gain the participant’s attention. Videos of the nurse, illustrations, and written information can be integrated in the same web page sequence. - Video testimonials of other patients, with an experience similar to the participants, are used. - Participants are asked about pain intensity and pain interference with activities to tailor the information on self-management behaviors and provide personalized feedback. Questions on the analgesics prescribed are also used to match content to participant needs. |
| **Health literacy strategies for printable educational manual** | **Design elements of the participant manual** |
| - Organize and structure the information to facilitate its understanding | - The cover of the manual includes the illustration of a lower extremity injury and pictures on what the intervention will focus on. - Only one behavior is presented per page. - Headings and subheadings are used to introduce the main message and divide the text. - Checklists include 7 items or less. - Blank spaces between the text and the illustrations and around the margins (2.5 cm) are used to keep the pages from being overwhelming. - The same format is used to present every non-web based session (4 to 7): 1) Session number with the date of occurrence, 2) Session objectives, 3) Review previous teachings, 4) How to reduce the medication for pain management, 5) Outline of the self-management behavior that needs to be mastered, 4) How to apply the self-management behavior, 5) Follow-up on activity and sleep objectives, and on the action plan for returning to previous activities. - Consistent frames and figures (i.e., arrows, target signs, and checklist signs) are used to highlight important information. A legend detailing the meaning of these frames and figures is provided at the beginning of the participant manual. |
| - Write actionable content | - The second-person pronoun is used to speak directly to the participant. - The benefits associated with the self-management behaviors described are presented. - Actions needed to apply the recommended self-management behaviors are clearly stated. - Headings and subheadings are formulated as questions whenever possible. - Examples on how to apply recommended self-management behaviors are provided. - Action plans or tables to promote reflection, to be filled out by participants, are included in each session. Examples on how to fill the tables are provided. - Reflective questions are provided to promote participant reflection on the implementation of self-management behaviors and to achieve their objectives. |
| - Communicate in clear and simple style | - A maximum of 15 words are used per sentence and paragraphs are limited to five sentences, as much as possible. - Font sizes are between 13 and 16 points in Arial style. Font size for headings is at least 2 points larger than for the main text. - The text is divided into bullet points. - Bold type is used to emphasize words or phrases instead of italics and underlining. - Scientific jargon and acronyms are avoided. - Photographs are used to illustrate self-management behaviors or real-life events. |
| - Consider culture | - The text used is neutral in terms of cultural background and gender. - Photographs illustrate various cultural backgrounds and genders. |
